# Total flavonoids of Oldenlandia diffusa (Willd.) Roxb. suppresses the growth of hepatocellular carcinoma through endoplasmic reticulum stress-mediated autophagy and apoptosis

**DOI:** 10.3389/fphar.2022.1019670

**Published:** 2022-11-29

**Authors:** Huan Chen, Xiaofei Shang, Huixin Yuan, Qianqian Niu, Jing Chen, Shumin Luo, Weihua Li, Xiuhui Li

**Affiliations:** ^1^ Integrated Chinese and Western Medicine Center, Beijing Youan Hospital, Capital Medical University, Beijing, China; ^2^ Beijing Institute of Hepatology, Beijing Youan Hospital, Capital Medical University, Beijing, China

**Keywords:** total flavonoids of *Oldenlandia diffusa* (Willd.), hepatocellular carcinoma, endoplasmic reticulum stress, autophagy, apoptosis

## Abstract

Hepatocellular Carcinoma (HCC) is one of the most common malignant tumors in the world. Although the current treatment methods for HCC are gradually increasing, its efficacy still cannot meet the medical needs of patients with liver cancer, and new and effective treatment strategies are urgently needed. The total flavonoids of *Oldenlandia diffusa* (FOD) are the main active components in *Oldenlandia diffusa*, which have anti-inflammatory, antioxidant and anti-tumor effects, but their mechanism of action in liver cancer is unclear. In this study, we examined the effect of FOD on HCC. Using both *in vitro* and *in vivo* models, we confirmed that FOD inhibited HCC proliferation and induced apoptosis and autophagy. Mechanistic studies have shown that FOD induces apoptosis and activates autophagy in HCC cells by inducing endoplasmic reticulum stress (ER stress) and activating the PERK-eIF2α-ATF4 signaling pathway. Taken together, our results suggest that FOD is a potential anticancer drug targeting ER stress for the treatment of HCC.

## Introduction

Hepatocellular carcinoma (HCC) remains a global challenge to human health, and its incidence has been increasing in recent years (Akinyemiju et al., 2017; Villanueva, 2019). In 2025, more than 1 million people will be affected by liver cancer, 90% of which will be hepatocellular carcinoma (Llovet et al., 2021). Currently, a variety of treatment options are available for HCC, and although drugs such as tyrosine kinase inhibitors (TKI)/antiangiogenic or immune checkpoint inhibitors (ICI) therapy are being used in the clinic, their efficacy still fails to meet the medical needs of HCC patients (Haber et al., 2021). As a result, new anti-HCC drugs are still urgently needed.

*Oldenlandia diffusa* (Willd.) Roxb is a plant commonly used in Chinese Tradition medicine and is widely distributed in the southern provinces of China (Hung et al., 2022). *Hedyotis diffusa Willd* is a kind of Chinese herbal medicine commonly used in clinic, which has anti-inflammatory, antioxidant and anti-proliferation effects (Luo et al., 2011; Li et al., 2020a; Hung et al., 2022). At present, more than 180 compounds have been found from *Hedyotis diffusa Willd*, including iridoid terpenes, flavonoids, anthraquinones, phenols and other substances (Chen et al., 2016a). The total flavonoids of *Hedyotis diffusa* (FOD) are the main active components of *Hedyotis diffusa Willd*, most of which are the derivatives of flavonol aglycone of kaempferol and quercetin (Chen et al., 2016a; Qian et al., 2021). FOD were found to have antitumor activity (Lee et al., 2011; Dong et al., 2014; Zhang et al., 2016). Previous studies have shown that the FOD is widely used to treat cancers of various types, including breast cancer (Ma et al., 2020), lung adenocarcinoma, lung cancer (Su et al., 2019; Huang et al., 2022), Prostate Cancer (Song et al., 2019a), Cervical Cancer (Qian et al., 2021) and HCC (Chen et al., 2012).

The endoplasmic reticulum (ER) is a central organelle which is responsible for the synthesis, folding, and posttranslational modification of membrane proteins and secreted proteins. Factors such as Hypoxia, High metabolic demand, and ROS overproduction can disrupt the protein-folding capacity of this organelle and lead to a state of ER stress that is characterized by the build-up of misfolded or unfolded proteins which leads to unfolded protein response (UPR) pathway activation (Chen and Cubillos-Ruiz, 2021). Under endoplasmic reticulum stress, three major branches of unfolded protein response (inositol-requiring enzyme 1α (IRE1α), protein kinase R (PRK)-like ER kinase (PERK), and activating transcription factor 6 (ATF6)) were activated to reprogram gene transcription, mRNA translation, and protein modifications to reduce the load of unfolded or misfolded proteins and restore protein homeostasis (Hetz et al., 2020). However, During the extreme ER stress condition, the UPR pathway can lead to cell death by inducing apoptosis and/or autophagy (Kim and Kim, 2018; Bhardwaj et al., 2020).

Although FOD can inhibit the proliferation and induce apoptosis of HCC cells (Huang et al., 2021) (Yang et al., 2021), the mechanism underlying its cytotoxicity to HCC cells and the role of reticulum (ER) stress remain unclear. Therefore, we investigated whether HDW induces autophagy and apoptosis through ER stress-signalling pathways in HCC cells.

## Materials and methods

### Chemicals and reagents

The crude total flavonoids of *Oldenlandia diffusa* (FOD) was prepared as follows: the raw materials were extracted with 80% ethanol, and then the extract was treated with lime cream and H_2_SO_4_ to exclude sediments and other compositions. After filtering, adding water, sealing, and sterilization, FOD was obtained. And each 1 ml of liquid contains 0.25 mg total flavonoids of *Oldenlandia diffusa* (Anhui Fengyang Keyuan Pharmaceutical Co. LTD., China, lot number 210514). 4-Phenylbutyric acid (4PBA) is purchased from MedChemExpress (Shanghai, China). Chloroquine (CQ) is purchased from Abmole (America). Rapamycin is purchased from MedChemExpress (America). Dulbecco’s Modified Eagle Medium (DMEM), fetal bovine serum (FBS), penicillin-streptomycin, and phosphate buffered saline (PBS) were purchased from Gibco. FITC Annexin V/PI Apoptosis Detection kit were purchased from Yeasen Biotechnology Co. Ltd. (Shanghai, China). Monodansylcadaverine (MDC), Lyso-Tracker Red (cat. no. L8010), Hoechst 33258 (cat. no. C0021) and DAPI (cat. no. C0065) were purchased from Beijing Solarbio (Beijing, China). Rabbit Anti-Phospho-PERK (Thr980) antibody, Goat Anti-rabbit IgG H&L/FITC antibody, Rabbit Anti-Mouse IgG-Fc/PE antibody, Mouse Cleaved caspase-3 antibody, Rabbit Anti-ATF4 antibody and Rabbit Anti-Phospho-EIF2S1 (Ser51) antibody were purchased from Beijing Boosen Biotechnology Co., LTD. LC3B, GAPDH, β-actin were purchased from Cell Signaling Technology (Boston, MA, United States). SQSTM1/p62 Mouse Monoclonal Antibody, CHOP antibody (Mouse mab), DDIT3/CHOP Rabbit Polyclonal Antibody and Ad-mCherry-GFP-LC3B were purchased from Beyotime Biotechnology (Shanghai, China).

### Cell line and culure

Human HCC cells HepG2, Hep3B, HCCLM3 and mouse hepatocellular carcinoma cells H22 were chosen for the following experiments, purchased from National Biomedical experimental cell resource bank (Beijing, China). The cells were incubated in DMEM medium supplemented with 10% FBS, 100U/ml penicillin-streptomycin and maintained at constant temperature 37°C in a sterile incubator with 5% CO_2_ as the normoxic condition. The digestion with 0.25% tryspin-EDTA was selected for use when the cells at about 70%–80% confluency. The cells whose growth cycle is in the logarithmic growth phase were selected for further experiments.

### Cck-8 assay

Hep3B, HepG2 and HCCLM3 cells were digested by 0.25% trypsin when the cells were in the logarithmic growth phase. The cells were collected for cell counting after centrifugation and were inoculated into each well of 96-well plate with 6 × 10^3^/well, 100 ul per well. This was cultivated for 24 h, and the cells were treated with FOD at different concentrations for 24 h and 48h, respectively. 10 μL CCK-8 detection reagent was added to each well and incubated for 2 h. The absorbance (A) of each well at 450 nm was measured using a microplate reader. Experiments were performed parallelly in triplicate.

### Cell apoptosis assay

Apoptosis was analyzed using an Annexin V-FITC/PI Apoptosis detection kit (YEASEN, Shanghai, China). 1.5 × 10^5^ HepG2 cells were inoculated in each well of the 6-well plates, were treated with FOD (12.5, 20, 25 μg/ml) for 24 h. Then the experiments were carried out according to the instructions. FACSVerse flow cytometer (BD Biosciences, San Jose, CA, United States) was used to detect the apoptotic cells. Data acquisition and analysis were performed using the Flowjo software (BD Biosciences, San Jose, CA, United States).

HepG2 cells were treated with FOD (12.5, 20, 25 μg/ml) for 24 h and then the original medium was removed and washed twice with PBS. 1ml of Hoechst 33258 (Solarbio) with a concentration of 5 μg/ml was added to each well of the six-well plate for 10 min at 37°C, and then PI dye solution was added to the final concentration of 15 μg/ml. The dye was stained for 10 min at 4°C, and then observed and photographed under a fluorescence microscope.

### Cell cycle detection

HepG2 cells treated with FOD (0, 12.5, 20, 25 μg/ml) for 24 h were collected and washed twice with ice-cold PBS. Cells were then fixed in 70% ethanol overnight at 4°C. With twice washing of PBS, cells were stained in solution with PI and RNAse according to the manufacturer’s operating instructions. A total of 30,000 events per sample were acquired by using flow cytometry (FACSCalibur, Becton Dickinson, San Jose, CA, United States), and cell cycle distribution were analyzed accordingly.

### Immunofluorescence detected by imaging flow cytometry

HepG2 were exposed to FOD (0, 12.5, 20, 25 μg/ml) for 24 h before harvest. Cleaved caspase-3, p-EIF2α and CHOP were stained with fluorescent labeled antibodies according to Feng, et al. reported method (Qian and Montgomery, 2015). Cells were then detected using a ImageStreamX MkII instrument (Amni, Luminex), and analyzed with IDEAS Software.

### Western blot analysis

We separated equal amounts of proteins from cells using sodium dodecyl sulfate-polyacrylamide gel electrophoresis and transferred them to polyvinylidene fluoride membranes. A 5% nonfat dry milk buffer was used to block membranes for 2 h at room temperature. Following blocking, membranes were incubated with appropriate primary antibodies at 4°C overnight, followed by 1 h at room temperature incubation with secondary antibodies. The proteins were visualised using SuperSignal West Pico PLUS reagents (ThermoFisher Scientific, United States), under the LAS-4000 mini luminescent image analyser (GE, PA, United States). Normalization was performed using the reference proteins. The results are displayed as the ratio of the target protein to internal reference normalized to the model group (the *y*-axis is the fold average of model values).

### ROS detection

HepG2 cells (1 × 10^6^ cells/well) were seeded in 6-well plates. After treatment with FOD for 24 h, cells were incubated with 1 ml of DCFH-DA (Reactive oxygen species assay kit, Solarbio, China) reagent at a concentration of 10 μM in each well of a six-well plate and incubated at 37°C for 20 min. The DCF fluorescence intensity was then immediately detected using a fluorescence microscopy.

### Animal study

All animal experiments were approved by the Experimental Animal Ethics Committee of Capital Medical University. Six-week-old female BALB/c athymic (Nu/Nu) mice were purchased from the Beijing Weitong Lihua Laboratory Animal Technology Co., LTD. and were acclimated to the institutional animal care facility for 1 week. Mice were injected subcutaneously with H22 cells (1 × 10^6^ per mouse) and were randomly divided into three groups: 6 mice in the control, 4 mice in the model, and 5 mice in the FOD (0.4 mg/kg/d). The FOD group was treated daily by intraperitoneal injection starting the day after tumor cell inoculation. Tumors take rate was 100%. Tumor growth was then measured for 2 days. The length and width of the tumors (mm) were measured three times a week using calipers. The tumor volume was calculated using formula (L ×  W^2^)  × 0.5, where L and W represent the length and the width, respectively.

### Statistical analysis

All experiments were repeated three times independently. Data were presented as means ± standard deviation (SD). Difference between groups was analyzed by one-way univariate analysis of variance (ANOVA) by Prism 8.0 software (San Diego, CA, United States), and the difference was considered significant when *p* value < 0.05 (marked as *).

## Result

### FOD halts progression of HCC *in vivo*

**FIGURE 1:**
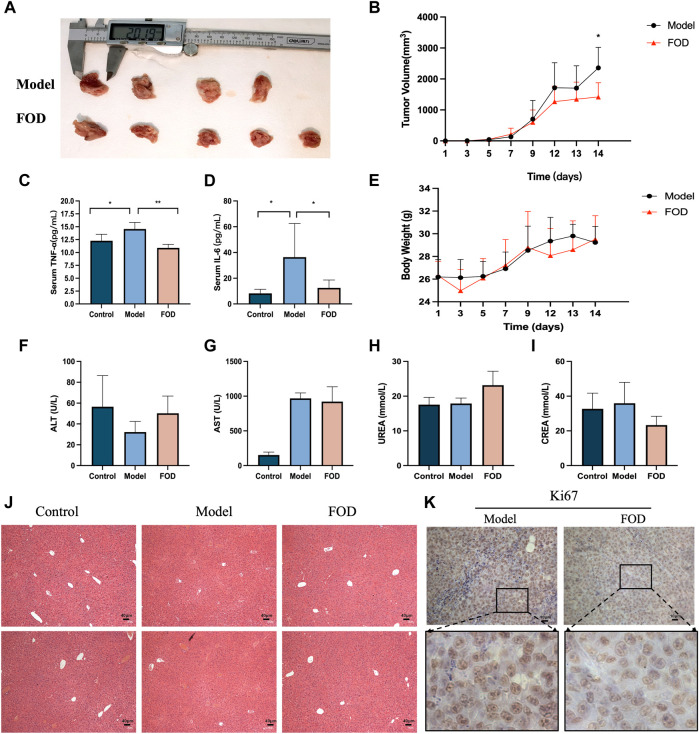
The inhibitor effects of FOD on the proliferation of HCC subcutaneous tumors. **(A)** Image of HCC subcutaneous tumors in model group and FOD group are shown. H22 cells were inoculated into BALB/C-nu mice, and the mice were randomly divided into two groups for model group (saline i.p) and FOD group (0.4 mg/kg/d i.p). **(B)** Tumor volume of mice in model group and FOD group at different time points. **(C,D)** Expression of IL-6 and TNF-α in blood of mice in control group, model group and FOD group. **(E)** Changes in body weight of mice in model and FOD groups at different time points. **(F–I)** Serum levels of ALT, AST, UREA and CREA in different groups of mice. **(J)** Hematoxylin and eosin (H&E) staining in livers of different groups of mice (scale bar, 40 μm). **(K)** Representative image of Ki67 immunohistochemical staining in tumor tissue (scale bar: 10 μm).

To investigate the effectiveness of FOD to inhibit HCC growth *in vivo*, 0.4 mg/kg/d FOD was administered to murine models injected with 1 × 10^6^ H22 cells. Studies have shown that FOD treatment can inhibit tumor proliferation. The tumor sizes in the groups of FOD treatment were significantly smaller model group, and the expression level of Ki67 was significantly decreased in the FOD group (Figures 1A,B,K). FOD has anti-inflammatory effect in mice, and the expressions of IL-6 and TNFα in serum of mice in FOD group were lower than those in model group (Figures 1C,D). The body weight, Alanine Aminotransferase (ALT), Aspartate transaminase (AST), UREA, CREA (Creatinine) and HE-stained tissue of liver in the FOD treatment groups were not significantly different from the model group (Figures 1E–J).

### FOD can induce endoplasmic reticulum stress and apoptosis of HCC *in vivo*

**FIGURE 2:**
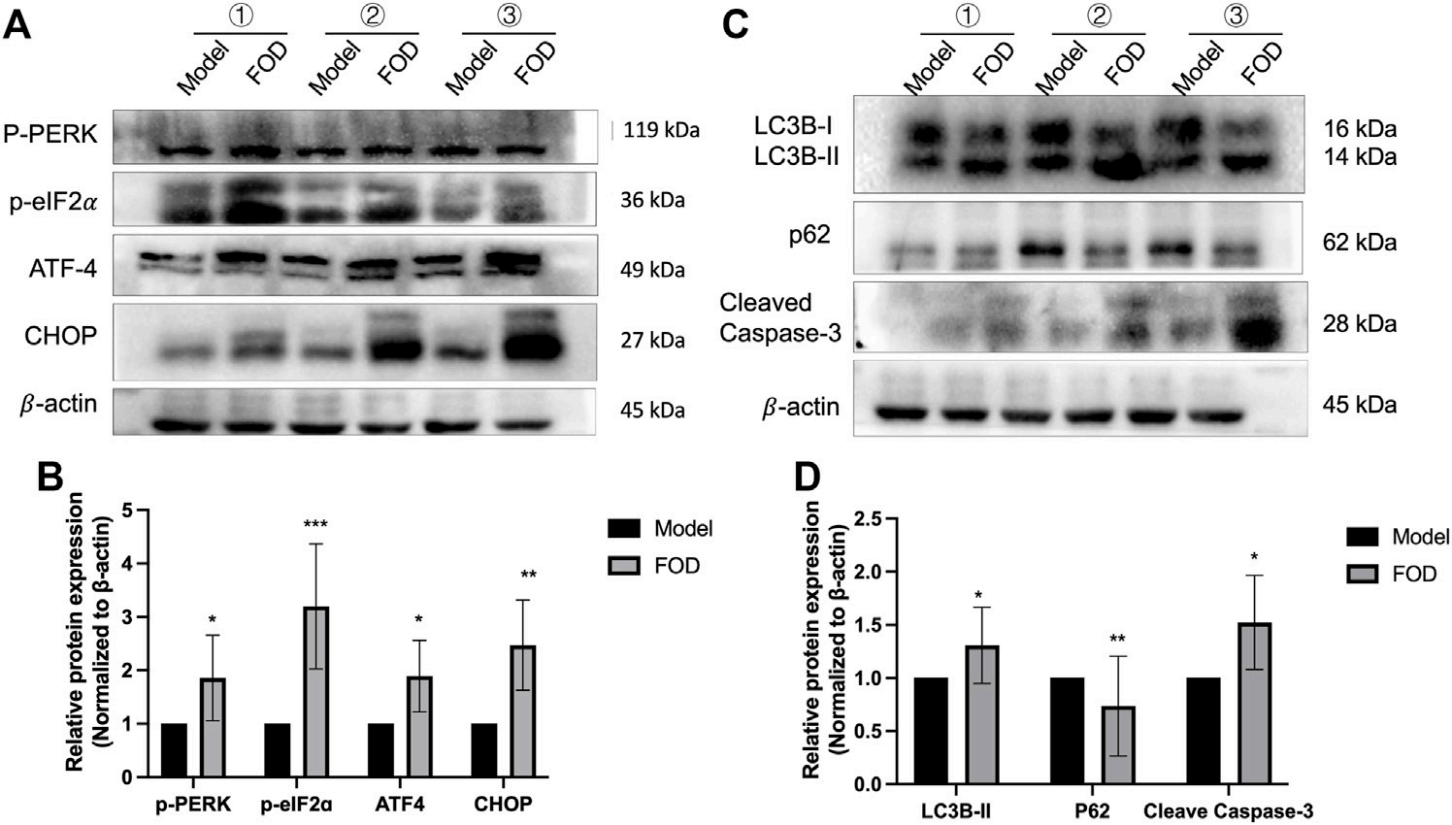
**(A,B)** Western blot analysis was used to evaluate the expression levels of p-PERK, P-EIF2α, ATF4 and CHOP proteins in tumor tissues; **(C,D)** LC3B-II, P62, and cleaved caspase3 proteins were examined using Western blot analysis in tumor cancer tissues. Values represent means ± SEM of at least 3 experiments. *p < 0.05, **p < 0.01, ***p < 0.001 compared with the control group.

To investigate in more detail the mechanism by which FOD inhibits HCC proliferation, we focused on endoplasmic reticulum stress. To further examine the FOD-induced ER stress, Western blotting was used to assess the expression of ER stress-related proteins. The results showed that FOD can significantly increase the expression levels of p-PERK, p-EIF2α, ATF4 and CHOP in tumor tissues of FOD group compared with model group (Figures 2A,B). CHOP is a pro-apoptotic transcription factor that stimulates cell apoptosis when ER stress is unresolved (Han et al., 2013; Hecht et al., 2021). Therefore, cleaved caspase-3 protein expression levels was detected, the results showed that the expression level of cleaved caspase-3 in FOD group was significantly higher than that in model group. Studies have highlighted that ER stress and autophagy are strictly interconnected (Kouroku et al., 2007; Jia et al., 2019; Fang et al., 2021). Next, we examined whether FOD triggers autophagy. As shown in Figures 2C,D, FOD significantly increased the protein expression level of autophagy-ralated molecules, including LC3-II and P62 in tumor tissues of FOD group.

### FOD can inhibit HCC cell proliferation *in vitro*

**FIGURE 3:**
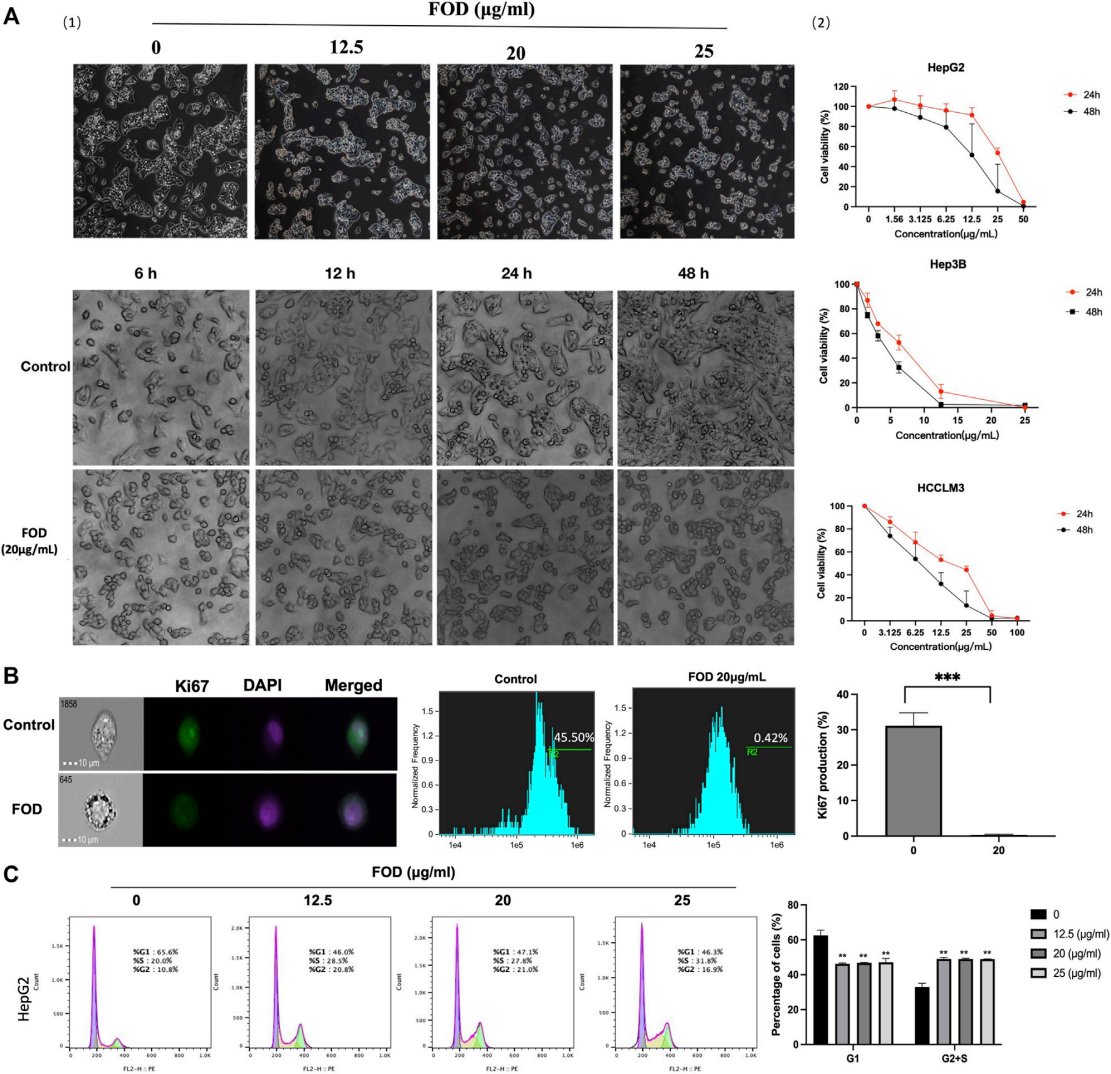
Total flavonoids of Hedyodium diffustidis (FOD) inhibits the proliferation of hepatocellular carcinoma (HCC) cells. **(A)** (1) HepG2 cells were treated with different concentrations of FOD for 24 h and treated with the same concentration of FOD for different drug durations. Cell status was observed under microscope (200 ×). (2) Human HCC cell lines (HepG2, Hep3B and HCCLM3) were treated with different concentrations of FOD for 24 h and 48h, respectively, and then the cell viability was determined by CCK-8. HepG2, HCCLM3, and Hep3B cell lines had 24 h IC50 values (Drug concentration at 24 h or 48 h inhibits cell growth by 50%) of 23.69, 12.89, and 5.75 μg/ml, respectively, and 48 h values of 13.05, 7.15, and 3.25 μg/ml **(B)** The expression of Ki67 in HepG2 cells treated with or without FOD (20 μg/ml) for 24 h was detected by imaging flow cytometry. **(C)** Cell cycle of HepG2 cells treated with different concentrations of FOD (12.5, 20, 25 μg/ml) for 24 h was detected by flow cytometry. After PI staining, flow cytometry analysis revealed that the number of G0/G1 phase cells decreased, while the number of cells in S and G2 phase increased after FOD treatment.

To determine the role of FOD in HCC, HepG2 cell was treated with FOD at different concentrations and for different lengths of time. The results showed that the cell morphology and growth of cells changed gradually with the increase of drug concentration and the extension of treatment time (Figure 3A). In order to further explore the effect of FOD on the proliferation of HCC cells,CCK-8 experiment were performed on HepG2, Hep3B and HCCLM3 cell lines. As shown in Figure 3A and Supplementary Figure S1A, FOD significantly inhibited the growth of HepG2, Hep3B, HCCLM3, and H22 in dose-and time-dependent manners. The expression of Ki67, a proliferation-related marker, was significantly decreased in HepG2 cells treated with FOD (Figure 3B). Furthermore, FOD induced cell cycle arrest in HepG2 cells (Figure 3C). These results suggest that FOD can inhibit HCC cell proliferation.

### FOD can induce apoptosis of HCC

**FIGURE 4:**
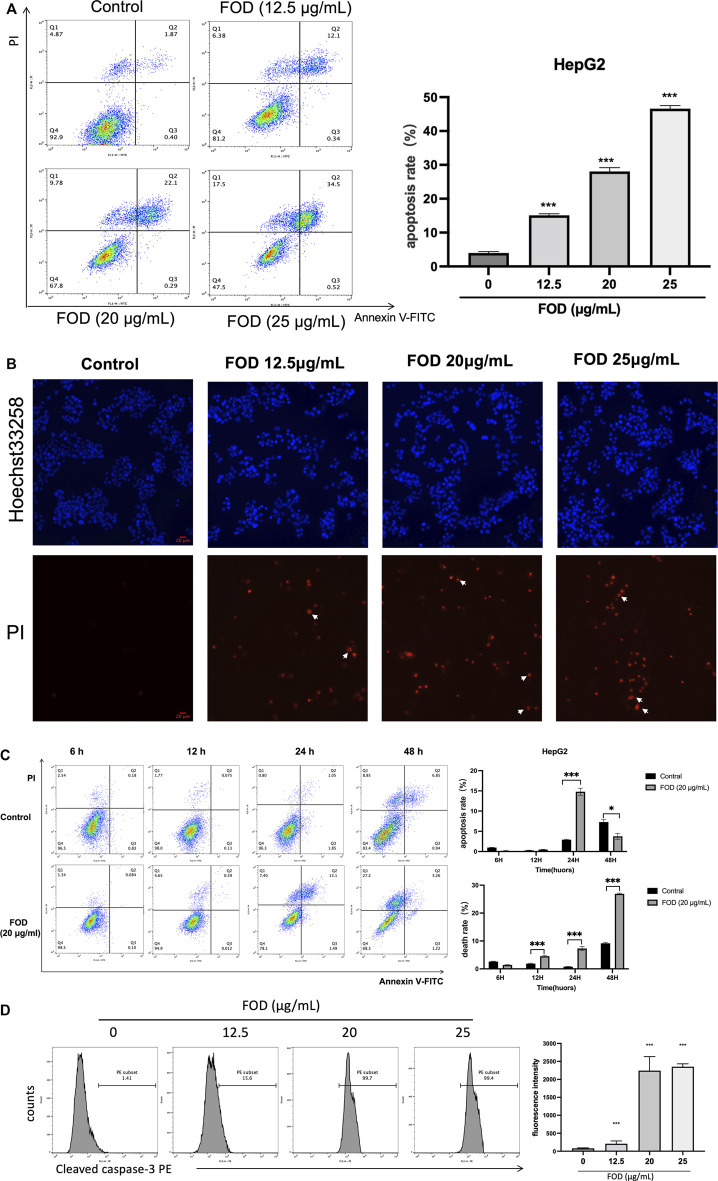
**(A)** Cells treated with different concentrations of FOD were harvested and annexin V and dead cells were determined by flow cytometry to quantitatively analyze apoptosis. **(B)** FOD-treated HepG2 cells were stained with Hoechst 33258/PI, and then observed under a fluorescence microscope. The white arrows show typical images of apoptosis. Scale bar: 20 μm. **(C)** HepG2 cells were treated with FOD (20 μg/ml). The cells were collected at different time points and the apoptosis and necrosis rates were detected by flow cytometry. **(D)** Flow cytometry was used to detect cleaved caspase-3 protein expression level in HepG2 cells treated with FOD for 24 h *p < 0.05, **p < 0.01, ***p < 0.001 compared with the control group. Data are expressed as the mean ± standard error of three experiments.

To explore the effect of FOD on the apoptosis of HCC cells, in the present investigation, HepG2 cells were treated with different concentrations of FOD (12.5, 20, 25 μg/ml) for 24 h. In addition, HepG2 cells were treated with FOD at a concentration of 20 μg/ml for 6 h, 12 h, 24 h, and 48 h. We utilized flow cytometry to analyse apoptosis of HCC cells, the results revealed that FOD can induced apoptosome occurrence in the HepG2 cells in dose-and time-dependent manners (Figures 4A,C). Supplementary Figure S1B shows that FOD could also induce apoptosis in mouse HCC cells H22. Hoechst33258/PI staining was used to detect apoptotic HepG2 cells. There were almost no apoptotic cells in the control group, but atrophic, hyperchromatic and pyknotic nuclei were observed in the FOD group (Figure 4B). We next studied cleaved caspase-3 expressions in HepG2 cells treated with different concentrations of FOD using Flow cytometry. As shown in Figure 4D, FOD treatment elevated the expression of cleaved caspase-3 protein.

### FOD can activate autophagy in HCC cells

**FIGURE 5:**
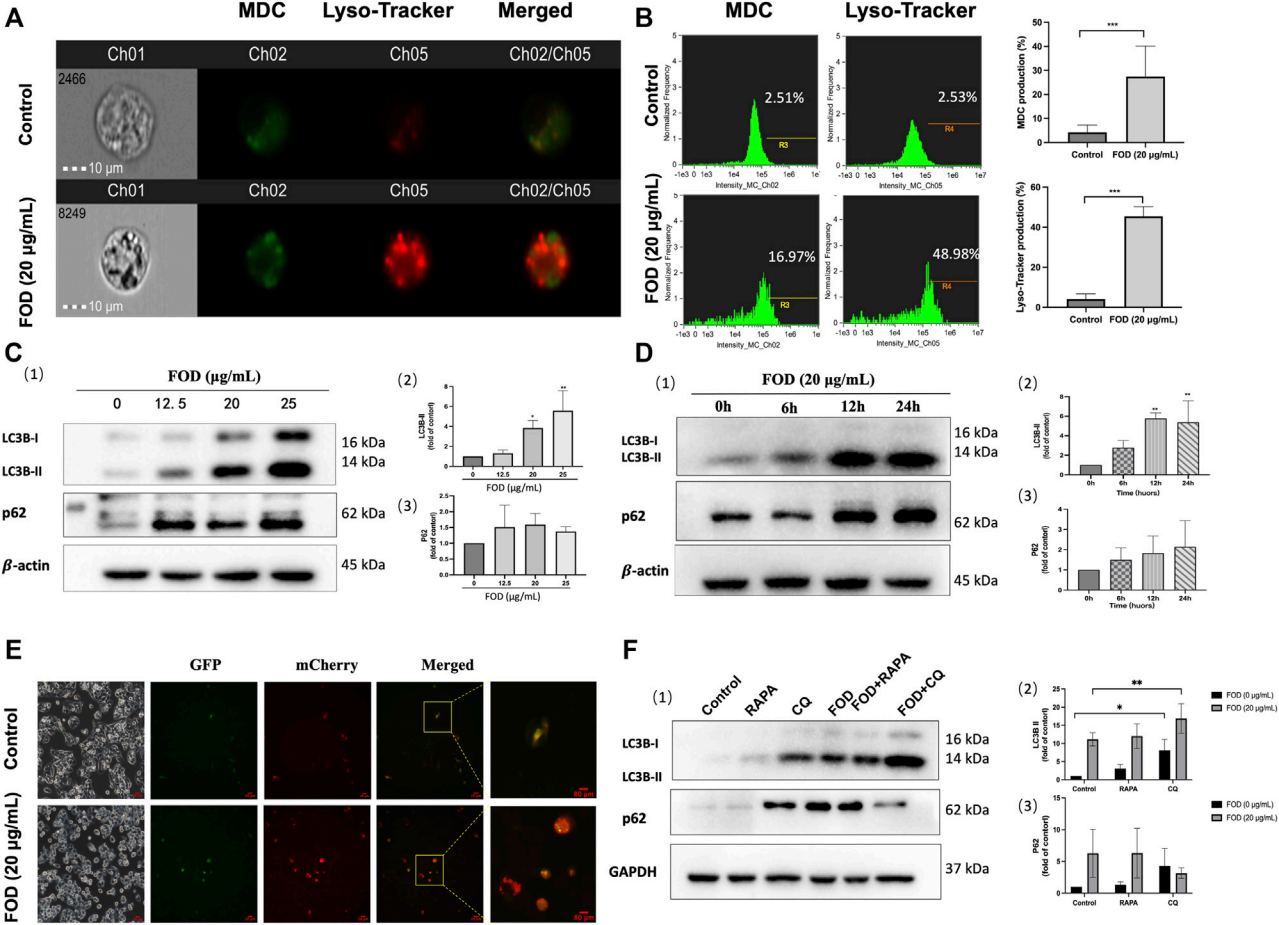
**(A,B)** HepG2 cells were treated with FOD (20 μg/ml) for 24 h and then stained with MDC and lyso-tracker Red. The fluorescence intensity was detected by flow cytometry. **(C)** HepG2 cells were treated with different concentrations of FOD for 24h, and the expressions of LC3B-II and P62 were detected by WB assay. **(D)** HepG2 cells were treated with FOD (20 μg/ml), and the expression of LC3B-II and P62 was detected at different time points. **(E)** HepG2 cells were transfected with mCherry-GFP-LC3 adenovirus and co-cultured with FOD (20 μg/ml) for 24 h. The cells were photographed under a fluorescence microscope. Scale bar: 20 μm. **(F)** The expression levels of LC3B-II and P62 in HepG2 cells were detected by RAPA and CQ alone or co-cultured with FOD (20 μg/ml) for 24 h. Data were expressed as mean ± standard deviation; *p < 0.05, **p < 0.01, ***p < 0.001.

In a subsequent study, we examined the effect of FOD on autophagy. By marking intracellular autophagosomes with MDC and Lyso-Tracker, we found that FOD could increase the generation of autophagosomes and the number of acid lysosomes in HCC cells (Figures 5A,B). We further confirmed the expression of autophagy signature protein LC3B-II by FOD through Western blotting experiment. As shown in Figures 5C,D, with the increase of FOD administration time and concentration, the expression of LC3B-II and P62 increased. In order to confirm the role of FOD in autophagy flux, HepG2 cells were transfected with the designed fusion protein mCherry-GFP-LC3B by adenoviral vector. Due to the superposition of GFP and mCherry signals, autophagosomes were marked as yellow. The autophagosome was marked red as a GFP signal quenched by low lysosomal pH (Song et al., 2019b). As shown in Figure 5E, most of the cells treated with FOD lost the GFP signal and retained the mCherry signal. However, in the control group, the signal expression of GFP and mCherry signal was very weak, indicating that the expression of autophagosome was very little and autophagy was not activated. Additionally, we introduced the autophagy promoter *Rapamycin* (RAPA) and autophagy inhibitor *Chloroquine* (CQ) to verify the effect of FOD on autophagy. As we know, CQ plays a role in inhibiting autophagy by decreasing autophagosome-lysosome fusion and blocking the autophagic flux (Mauthe et al., 2018). HepG2 cells were pretreated with RAPA and CQ for 2 h, and then incubated for 24 h with cells in the presence or absence of FOD. According to the results (Figure 5F), LC3-II levels were higher when FOD and CQ were combined than when CQ was used alone. Therefore, FOD treatment increases autophagy-related membrane synthesis and activates the process, which is similar to the results of classical experiments that detect autophagy flux (Klionsky et al., 2021). Overall, our findings suggest that FOD can activate autophagy.

### Induced apoptosis and autophagy by FOD are mediated by ER stress

**FIGURE 6:**
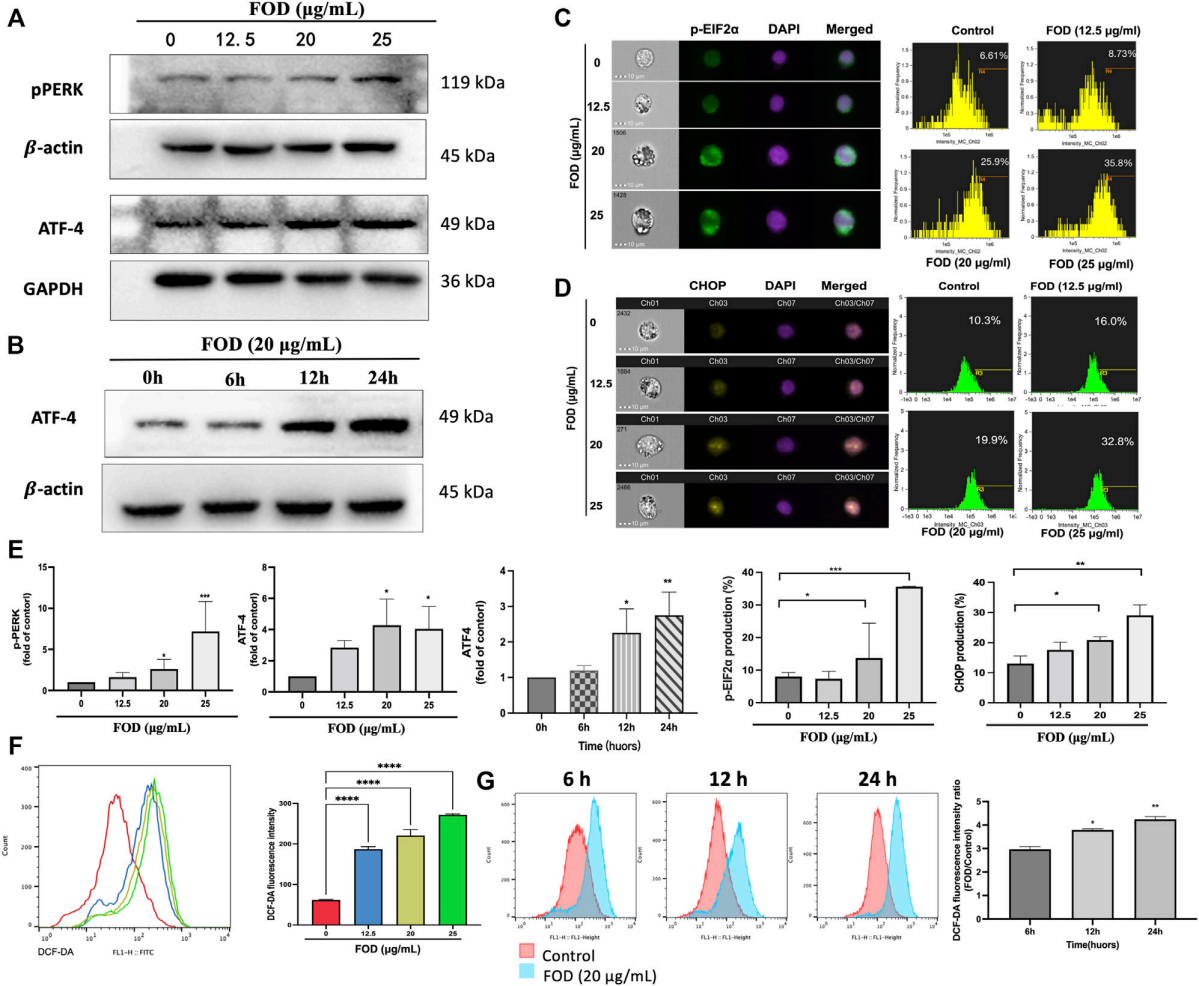
**(A)** HepG2 cells were treated with different concentrations of FOD for 24h, and the levels of p-PERK and ATF-4 were detected by Western blotting. **(B)** HepG2 was treated with FOD (20 μg/ml), and the expression level of ATF4 at different time points was detected by Western blotting. **(C,D)** The expression levels of p-EIF2α and CHOP protein in HepG2 cells treated with different concentrations of FOD for 24 h were detected by imaging flow cytometry. **(E)** The histogram shows the quantification results of the expression levels of p-PERK, ATF4, P-EIF2α and CHOP. **(F,G)** A concentration and time-dependent manner was observed on FOD induced ROS generation. Data were expressed as mean ± standard deviation; *p < 0.05, **p < 0.01, ***p < 0.001.

Numerous studies have found a link between autophagy and ER stress (Yu et al., 2019; Zhang et al., 2021a; Chipurupalli et al., 2021; Gámez-García et al., 2021; Jahangiri et al., 2022), so we focused on ER stress in this study. p-PERK and p-EIF2α are ER stress-related proteins, and the results show that FOD increases their expression (Figures 6A,C; Supplementary Figure S1C). ATF4 is a key link between ER stress response pathway and autophagy gene expression, because it directly binds to the promoters of several autophagy genes (MAP1LC3B, ATG12 and BECN1) and upregulates their expression (Luhr et al., 2019; Muñoz-Guardiola et al., 2021). Western blot analysis showed that the expression of ATF4 increased dose-dependently and time-dependently after FOD treatment (Figures 6A,B). Study have reported that ATF4 can promote cell apoptosis by regulating the expression of CHOP, which encodes pro-apoptotic protein (Park et al., 2022). By imaging flow cytometry, CHOP expression in HepG2 cells treated with different concentrations of FOD was detected. The results showed that a higher level of CHOP expression was observed after exposure to FOD (Figures 6D,E). Previous studies have shown that excessive activation of ERO1α by CHOP during ER stress increases ROS production (Hetz, 2012). ROS were detected in HepG2 cells treated with FOD at different concentrations and for different periods of time. As shown in Figures 6F,G, FOD could increase the ROS level in HCC cells in a concentration and time-dependent manner. Therefore, we hypothesized that ER stress is important for FOD-induced apoptosis of HCC cells.

**FIGURE 7:**
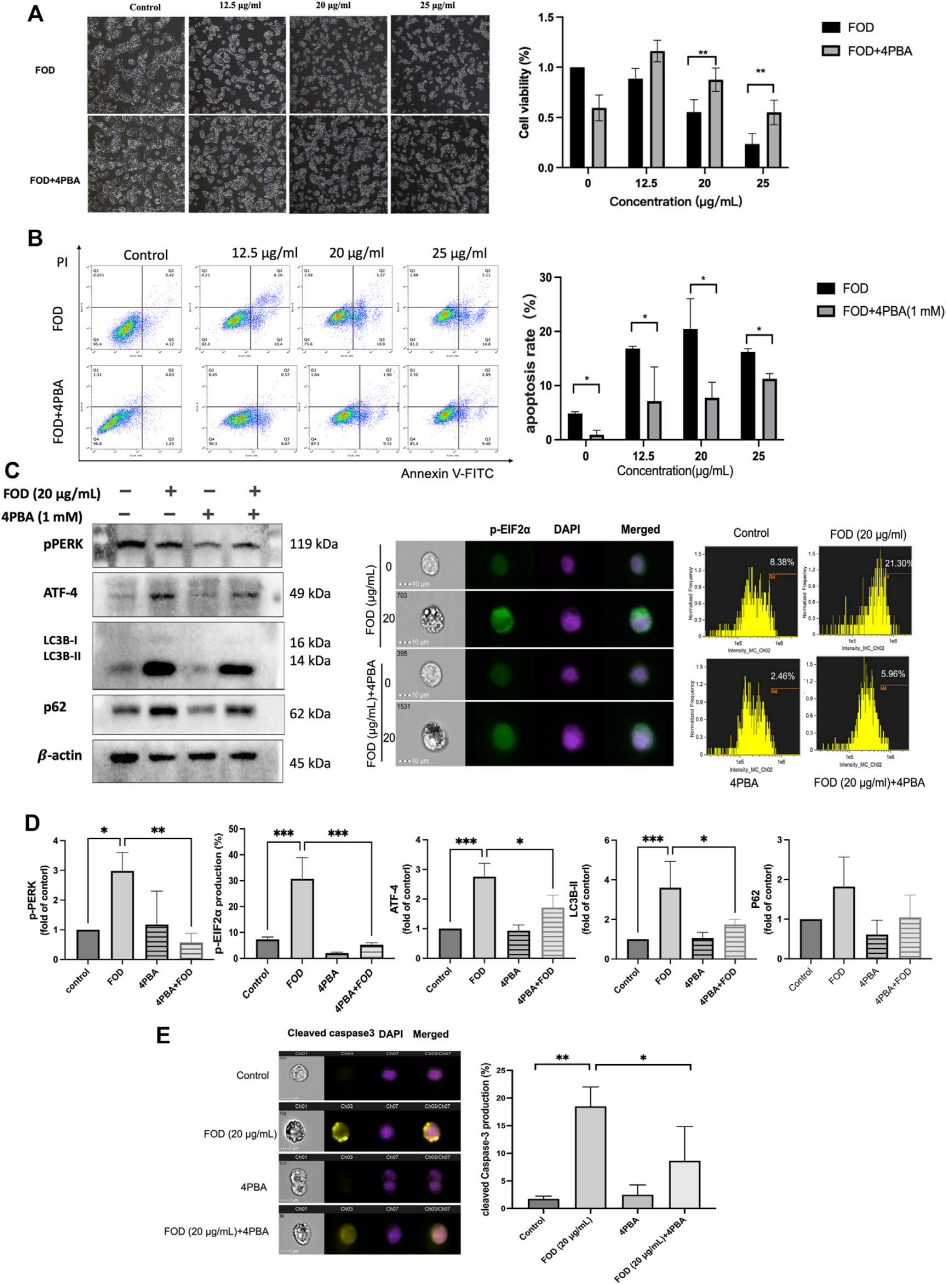
**(A,B)** FOD was incubated with HepG2 for 24 h in the presence or absence of 4PBA (1 mM) pretreatment, and cell viability was analyzed by CCK-8 assay. The apoptosis rate was detected by flow cytometry. **(C,E)** FOD was incubated with HepG2 for 24 h in the presence or absence of 4PBA (1 mM) pretreatment. The expression levels of p-PERK, ATF4, LC3B-II, P62, p-EIF2α and Cleaved caspase-3 were detected by Western blotting or flow cytometry. **(D)** Quantification of expression levels of p-PERK, ATF4, LC3B-II, P62, p-EIF2α and Cleaved caspase-3. Data were expressed as mean ± standard deviation; *p < 0.05, **p < 0.01, ***p < 0.001.

To further validate the effect of ER stress on FOD-induced apoptosis and autophagy activation, 4-Phenylbutyric acid (4PBA), a putative ER stress inhibitor, was used to demonstrate. We pretreated HepG2 cells with 4PBA(1 mM) for 2 h and then treated cells with FOD. The results showed that compared with the group treated with FOD alone, the FOD group treated with 4PBA increased the survival rate of HepG2 cells and decreased the apoptosis rate (Figures 7A,B). These results indicated that FOD-induced apoptosis was mediated by ER stress. WB and flow cytometry results showed that pretreatment of HCC cells with 4PBA not only reduced the expression of ER stress-related proteins induced by FOD, but also decreased the expression of autophagy marker LC3B-II (Figures 7C,D). The expression of cleave-caspase3, an apoptosis-related protein, was further detected in HCC cells after inhibition of PERK/EIF2α/ATF4 pathway. As shown in Figure 7E, inhibition of ER stress pathway could reduce the expression of Cleave-Caspase3 after FOD treatment. Overall, these results suggest that FOD induces apoptosis and autophagy in HCC cells by inducing ER stress.

## Discussion

*Hedyotis diffusa Willd* is a commonly used Chinese herbal medicine with various pharmacological activities such as anti-tumor, anti-inflammatory, antioxidant and immunomodulatory (Dong et al., 2014) (Chen et al., 2016b) (Gao et al., 2016) (Kuo et al., 2015a). It has been used to treat various diseases for thousands of years (Meng et al., 2013). FOD are the main active components of *Hedyotis diffusa Willd* (Chen et al., 2016a; Qian et al., 2021). Previous studies have found that FOD can inhibit the proliferation of HCC cells, block the G0/G1 cell cycle, induce apoptosis, and down-regulate AKT/mTOR and CDK2-E2F1 pathways in HCC cells, and inhibit the growth of mouse xenograft tumors (Chen et al., 2012; Chen et al., 2020; Huang et al., 2021). Our study sought to verify that FOD can induce apoptosis and autophagy by activating the ER stress pathway in hepatocellular carcinoma. In this study, FOD reduced the volume of subcutaneous tumor in mice and inhibited the proliferation, which was consistent with the previous results obtained by Huang et al. in the HepG2 xenograft subcutaneous tumor mouse model (Huang et al., 2021). At the same time, we also found that FOD reduced the inflammatory indicators (IL-6, TNFα) in the blood of mice. Previous studies have found that FOD can exert anti-inflammatory effects by activating NF-κB and MAPK pathways (Chen et al., 2016c). *In vitro* experiments, we found that FOD reduced the survival rate of HCC cells, inhibited the proliferation of HepG2 cells by inducing cell cycle arrest, and induced apoptosis of HCC, which was consistent with previous findings (Lee et al., 2011; Chen et al., 2012; Kuo et al., 2015b; Lin et al., 2015).

In further experiments, we observed that FOD could induce autophagy activation in HCC. Although P62, as a substrate for autophagy, is degraded by lysosomal dependent autophagy pathway in most cases (Babu et al., 2005; de Wet et al., 2021). However, our studies showed that FOD did not decrease P62 expression *in vitro*. The reason for this may be that P62 is not always decreased during autophagy. In some cases of increased autophagy flux, the overall number of P62 was unchanged or increased (Toepfer et al., 2011; Zheng et al., 2011). Alterations in P62 may be therapeutic specific, such that chemotherapy-induced autophagy increases LC3-II without altering P62, whereas radiation-induced autophagy increases LC3-II and reduces P62 in murine breast cancer cells overexpressing ERBB2/her2 (Toepfer et al., 2011; Zheng et al., 2011; Aqbi et al., 2018). Studies have induced mouse embryonic fibroblast (MEF) cells by Rapamycin, and the results showed that the expression of P62 was basically unchanged after the treatment of high and low concentrations of Rapamycin (de Wet et al., 2021). Thus, P62 expression alone cannot be used to assess the effect of drugs on autophagy. As a result, FOD’s effect on autophagy was examined from a variety of perspectives. According to flow cytometry imaging, FOD increased autophagosomes and acid lysosomes in cells. According to the results of Western Blot, FOD could increase LC3B-II expression in a concentration-time-dependent manner. The colocalization of mCherry and GFP in mCherry-GFP-LC3B-expressing cells also confirmed that FOD promotes autophagy flux smoothly. Finally, using FOD alone or in combination with an autophagy inhibitor/promoter, we confirmed that FOD activated autophagy in HCC cells and promoted autophagy flux. In conclusion, our experiments verified that FOD could activate autophagy in HCC cells from many aspects.

As we know, autophagy is an evolutively conserved catabolic degradation process in cells, in which cytoplasmic macromolecules, aggregates, and damaged organelles are transported to lysosomes and digested by lysosomal hydrolases to produce nucleotides, amino acids, fatty acids, sugars, and ATP, which are eventually recycled into cytosols (Li et al., 2020b). Previous studies have reported a close relationship between autophagy activation and ER stress response (Song et al., 2018; Bhardwaj et al., 2020). ER stress can effectively induce autophagy, because malignant tumor cells need to reuse their organelles to maintain growth. Autophagy also counteracts ER stress-induced ER expansion and enhances cell viability and non-apoptotic death (Lin et al., 2019). Our study found that FOD could induce apoptosis in HCC cells by activating PERK-EIF2α-ATF4 signaling pathway. Studies have shown that ATF4 can activate the transcription of 29 kDa bZIP transcription factor called CCAAT/enhancer binding protein homolog (CHOP) (Rozpedek et al., 2016) and increase the expression of CHOP. CHOP is a well-known mediator of ER stress-mediated cell death, which activates a large number of pro-apoptotic factors and aggravates oxidative stress (Bhardwaj et al., 2020). This is consistent with the results we observed after treating HCC with FOD *in vitro* and *in vivo*. Meanwhile, the activation of autophagy may be related to cell death (Denton and Kumar, 2019). Cells undergoing cell death have increased the number and size of autophagic vesicles/vesicles compared to cells undergoing starvation induced autophagy (Arakawa et al., 2017), suggesting that excessive activation of autophagy may promote cell death. In fact, cell death is also increased when the feedback mechanisms that inhibit autophagy are disrupted (Füllgrabe et al., 2013).

In previous studies, Hedyotis Diffusa Polysaccharide Extract could induce endoplasmic reticulum stress in kidney cancer HEP-2 cells to mediate cell apoptosis (Zhang et al., 2021b). To date, no experimental verification hypothesis has been proposed that FOD leads to sustained ER stress and plays an anti-HCC mechanism. Our study confirmed that FOD can induce apoptosis and autophagy in HCC by inducing ER stress response and activating PERK-EIF2α-ATF4 signaling pathway.

In conclusion, we report that FOD can activate autophagy and induce apoptosis in HCC through ER stress. Traditional Chinese medicine (TCM) is a great treasure house, and further in-depth development may provide new options for the treatment of HCC. For future consideration, different mouse strains can be added in the subsequent studies, and different HCC modeling methods can be used for experiments, so as to further clarify the effect of FOD on tumors in different HCC models.

## Data Availability

The original contributions presented in the study are included in the article/supplementary material, further inquiries can be directed to the corresponding authors.
