# Supplementary material for: Total flavonoids of Oldenlandia diffusa (Willd.) Roxb. suppresses the growth of hepatocellular carcinoma through endoplasmic reticulum stress-mediated autophagy and apoptosis
Source: Front Pharmacol. 2022 Nov 29;13:1019670. doi: 10.3389/fphar.2022.1019670 (PMC9745173; doi:10.3389/fphar.2022.1019670)
Supplement: Supplementary file 1 [file Table1.DOCX]

**Supplementary Table 1. IC_50_ of FOD on four HCC cell lines**.

|  | IC50 (μg/ml) | |
| --- | --- | --- |
|  | 24h | 48h |
| HepG2 | 23.69 | 13.05 |
| HCCLM3 | 12.89 | 7.15 |
| Hep3B | 5.75 | 3.25 |
| H22 | 17.6 | 3.75 |
