# Supplementary figures and images for: Total flavonoids of Oldenlandia diffusa (Willd.) Roxb. suppresses the growth of hepatocellular carcinoma through endoplasmic reticulum stress-mediated autophagy and apoptosis
Source: Front Pharmacol. 2022 Nov 29;13:1019670. doi: 10.3389/fphar.2022.1019670 (PMC9745173; doi:10.3389/fphar.2022.1019670)

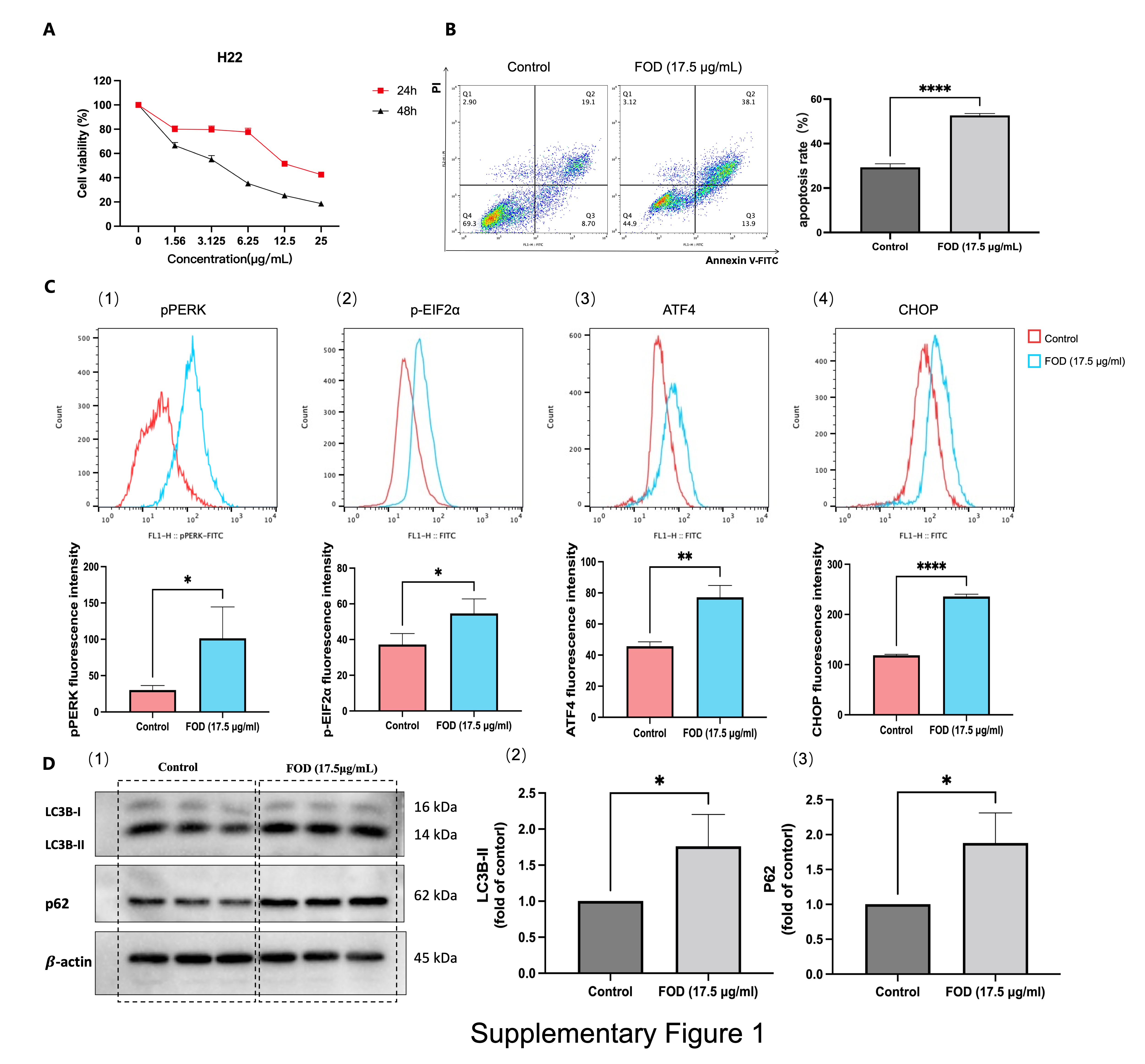

Supplement: Supplementary file 2 [file Image1.JPEG]
